# Epidemiology and containment of the first Marburg virus disease outbreak in Ethiopia in 2025: A retrospective descriptive study

**DOI:** 10.1016/j.gloepi.2026.100276

**Published:** 2026-07-01

**Authors:** Hailemariam Mamo Hassen, Amelmasin Faris Ibrahim

**Affiliations:** a Department of Public Health, College of Medicine and Health Science, Dire Dawa University, Dire Dawa, Ethiopia; b Department of Anesthesia, College of Medicine and Health Science, Dire Dawa University, Dire Dawa, Ethiopia

**Keywords:** Marburg virus disease, Ethiopia, Outbreak response, Reproduction number, Medical logistics

## Abstract

**Background:**

In November 2025, Ethiopia confirmed its first Marburg Virus Disease (MVD) outbreak in Jinka, marking a significant geographical expansion of the virus with a 64% case fatality rate. This study characterizes the transmission dynamics of the 2025 MVD outbreak in Ethiopia and evaluates the impact of the national public health containment strategy on the epidemic trajectory.

**Methods:**

We conducted a retrospective descriptive analysis using surveillance and laboratory data from November 14 to December 30, 2025. Transmission intensity was quantified using basic (R_0_) and effective (R_t_) reproduction numbers. We further evaluated the deployment of the investigational cAd3-Marburg vaccine and the feasibility of drone-assisted ultra-cold chain logistics under the national Evidence Generation during an Emergency (EGE) framework.

**Findings:**

The outbreak involved 14 confirmed cases and 9 fatalities (CFR: 64%). The initial R_0_ was 2.34, driven by nosocomial and religious clusters. Following the activation of the Incident Management System, diagnostic testing increased by over 1000%, and 2500 vaccine doses were deployed to high-risk groups. The use of drone logistics was observed to support the −80 °C cold chain for deliveries in remote areas. R_t_ fell below 1.0 within 14 days of formal intervention.

**Interpretation:**

The findings are consistent with the hypothesis that the combination of rapid diagnostic scaling decentralized incident management and technological integration were temporally associated with the truncation of MVD transmission chains. The integrated approach of decentralized management and rapid technological deployment offers a framework for viral hemorrhagic fever preparedness in similar resource-limited settings.

## Introduction

The emergence of Marburg Virus Disease (MVD) in Ethiopia in late 2025 represents a significant expansion of the virus's known geographic range [1]. MVD is caused by the Marburg virus (MARV), is characterized by its sudden onset, aggressive pathogenesis, and high lethality, with historical case fatality rates reaching as high as 90% in some outbreaks [2], [3]. Marburg virus (MARV) is a high-consequence zoonotic pathogen characterized by rapid onset and high case-fatality rates, presenting significant challenges for containment in resource-limited settings on November 14, 2025, the Ethiopian Ministry of Health (MoH) officially declared the country's first documented MVD outbreak in Jinka Town, South Ethiopia Regional State [4], [5], [6], [7]. The outbreak required a response from national clinical systems and had implications for regional health security, given Jinka's proximity to the borders of Kenya and South Sudan [8].

The 2025 Ethiopian outbreak was characterized by its origin at a commercial construction site in Jinka Town [1]. The Commercial Bank of Ethiopia (CBE) building, under construction since 2017, provided an accidental habitat for *Rousettus aegyptiacus* colonies, creating a direct zoonotic interface for workers [[1], [9]]. The identification of this spillover point preceded the mapping of the subsequent Jinka-Church-Hospital transmission axis. While a preliminary alert on this outbreak was previously published [1], this study characterizes the transmission dynamics of the 2025 MVD outbreak in Ethiopia and evaluates the impact of the national public health containment strategy on epidemic trajectory [10].

MVD was first identified in 1967 when laboratory personnel in Germany [11], and Yugoslavia were infected while processing tissues from monkeys imported from Uganda [12]. Since the first identification of the virus, there have been sporadic outbreaks reported primarily in Central and East Africa [[13], [14], [15], [16], [17], [18]]. Notably, neighboring Kenya has a documented history of MVD, including the 1980 Mount Elgon case and subsequent infections in 1987, which highlight the long-standing ecological presence of the virus in the region [19]. More recent patterns demonstrate an increasing frequency of spillover, with notable outbreaks in Ghana (2022), Tanzania (2023), Equatorial Guinea (2023), and Rwanda (2024). [[13], [14], [15], [16], [17], [18]]. The 2004–2005 outbreak in Uige, Angola, remains the largest in history, reporting 252 cases and a 90% fatality rate. Recent patterns demonstrate increasing frequency of spillover, with notable outbreaks in Ghana (2022), Tanzania (2023), Equatorial Guinea (2023), and Rwanda (2024) [[14], [15], [16]]. The lethality of MVD is profound [20]. Case fatality rates have historically varied depending on the specific outbreak, virus strain, and availability of advanced supportive care, ranging widely from 24% to 88% [[21], [22]]. Specific outbreaks have documented mortality rates as high as 100% [[20], [22], [23]]. The estimated average global CFR for MVD outbreaks is approximately 61·9% [20]. Analysis of recent outbreaks across Africa, including Equatorial Guinea (88%), Tanzania (67%), and Ghana (67%), confirms the consistently high mortality associated with this pathogen [[14], [15], [16]]. Despite this high mortality burden, there are currently no licensed vaccines or specific antiviral therapeutics available for MVD [[14], [15], [16]], rendering comprehensive public health measures and extensive diagnostic surveillance., alongside timely supportive medical care (including early rehydration), the central pillars of outbreak control [24].

The 2024 Rwanda outbreak provides a critical contemporary benchmark for Ethiopia's 2025 response. In Rwanda, a cluster of 66 cases was traced to a miner exposed to bat-inhabited tunnels [[13], [18], [24]]. Rwanda reported a CFR of 23%, demonstrating that urban transmission can be contained through rapid multidisciplinary interventions. In contrast, Ethiopia's 2025 outbreak exhibited a significantly higher CFR of 64%, highlighting the challenges of managing VHFs in remote areas where access to advanced critical care and hemodynamic monitoring is limited [[1], [10]].

While a preliminary clinical alert was issued during the early phase of the 2025 Jinka outbreak, a comprehensive scientific evaluation of the transmission dynamics and the specific technological interventions employed has not yet been documented. The emergence of Marburg Virus Disease (MVD) in Ethiopia in late 2025 poses a critical public health challenge. While the clinical characteristics of the outbreak have been noted, there remains a critical need to evaluate the effectiveness of the national containment response. This study addresses this gap by focusing on two primary research questions:

1. What were the transmission dynamics of the 2025 MVD outbreak, and how did they evolve in relation to the national public health containment strategy?
2. What is the feasibility of deploying investigational medical countermeasures, specifically the cAd3-Marburg vaccine and drone-assisted cold-chain logistics, within an emergency framework in a resource-limited setting?

This study addresses this gap by providing a detailed retrospective analysis of the outbreak's progression through three distinct clusters (industrial, religious, and nosocomial). Furthermore, it offers anevaluation of the Ethiopian strategy and the feasibility of maintaining ultra-cold chain logistics via autonomous drone systems in resource-limited settings. By quantifying the shift in the effective reproduction number (Rt) relative to these interventions, it contributes new evidence to the global framework for managing high-consequence viral hemorrhagic fevers. This paper analyzes the epidemiological trajectory of the 2025 outbreak, focusing on how the deployment of centralized surveillance and rapid logistics supported national containment efforts.

## Methods

### Study design and surveillance period

This study is a retrospective descriptive analysis utilizing epidemiological and laboratory line-list data from the Ethiopian Public Health Institute (EPHI) and the Ministry of Health (supplementary file1). The study period covers the immediate response phase from the initial declaration on November 14, 2025, through December 30, 2025. We focused on laboratory-confirmed cases in Jinka Town and analyzed secondary transmission chains in the Sidama Region (Hawassa) and Dassanech Woreda [[22], [25], [26]].

### Case definition and laboratory confirmation

A confirmed case was defined as any person, irrespective of symptoms, with laboratory confirmation of MARV infection via Real-Time Reverse Transcription Polymerase Chain Reaction (rRT-PCR) [11]. Molecular assays targeted the NP and VP40 genes to ensure high sensitivity and specificity. Suspected samples (whole blood or oral swabs) were collected in Viral Transport Media (VTM) and transported via a triple-packaging cold chain system maintained at 2 °C to 8 °C to the national reference laboratory in Addis Ababa and the mobile laboratory in Jinka. To achieve rapid containment, laboratory shifts were expanded to 24-h cycles, reducing the turnaround time (TAT) to under 12 h for high-risk contacts.

### Data collection and epidemiological analysis

This dataset comprises primary, unpublished surveillance records from EPHI, distinct from the preliminary clinical aggregates reported in earlier situational alerts [[26], [27]]. The analysis quantified the total number of confirmed cases, deaths, and recoveries, and calculated the Case Fatality Rate (CFR). Further analysis focused on the demographics, geographic distribution of cases across South Ethiopia and Sidama, and the specific transmission pathways identified. Response metrics were also compiled, tracking the public health effort. These metrics included the total number of laboratory tests conducted and the total number of individuals identified and placed under active follow-up as contacts. Qualitative information regarding key transmission chains, including details of the index case, nosocomial spread, and community engagement challenges, was compiled from field investigation summaries and external partner situation updates. This study represents a significant analytical advancement over the preliminary epidemiological alerts issued during the active phase of the 2025 outbreak.

### Mathematical modeling of transmission dynamics

To quantify transmission intensity, we utilized two distinct analytical frameworks, acknowledging that $R_{0}$ and $R_{t}$ are derived from different mathematical assumptions and temporal windows and should not be directly compared as equivalent measures. $R_{0}$ and $R_{t}$ are not directly comparable metrics: $R_{0}$ represents transmissibility at the intrinsic baseline under conditions of a fully susceptible population and no active control measures ($R_{0}=e^{rT_{c}}$) [[19], [28]], while $R_{t}$ captures dynamic, time-varying transmissibility conditional on the prevailing susceptibility landscape and the cumulative effect of sequential interventions ($R_{t}=\frac{I_{t}}{\sum_{s=1}^{t}I_{t-s}g\left(s\right)}$) [29]. Any apparent numerical difference between $R_{0}$ and a given $R_{t}$ estimate therefore reflects the combined biological, behavioural, and programmatic changes between the two reference periods, rather than a simple before–after intervention effect.

### Policy and ethical framework for investigational vaccine use

In the absence of licensed medical countermeasures, the Ethiopian MoH authorized the deployment of the investigational cAd3-Marburg vaccine under a Phase 2 rapid-response clinical trial [[1], [10], [30], [31]]. This deployment was conducted under the national Evidence Generation during an Emergency (EGE) framework, which balances the ethical requirement to protect frontline personnel against the scientific imperative for data collection [10]. The trial utilized a two-cohort protocol:

• Cohort A (Priority Group): Limited to high-risk healthcare workers and direct contacts of infected persons within the 21-day incubation period.
• Cohort B (Randomized Group): Other frontline workers and contacts randomized to receive the vaccine either on Day 1 or Day 22 of enrollment.

This study does not evaluate the clinical efficacy of the cAd3-Marburg vaccine or drone logistics but rather describes their deployment as part of the operational containment strategy. The impact of these interventions is assessed through their temporal association with the decline in the effective reproduction number ($R_{t}$).

The epidemiological and response dynamics of the outbreak were evaluated using several primary study variables. The Case Fatality Rate (CFR) was calculated as the percentage of total confirmed deaths relative to total confirmed cases, providing a measure of the outbreak's lethality. The Serial Interval (T_s_), representing the time between symptom onset in primary and secondary cases, was estimated using detailed contact tracing narratives from identified clusters. Finally, Response Scaling was quantified by tracking the expansion of daily rule-out testing volume over the duration of the intervention period.

## Results

### Epidemiological narrative and transmission dynamics

The cumulative daily suspected laboratory tests from November 17 to December 30/12/2025 showed a clear pattern of exponential growth in testing activity. Initially, the cumulative number of tests increased to 122 by November 28; subsequently, surveillance volume increased to a total of 3541 cumulative tests by December 30, 2025 (Table 1).

**Table 1:** Comprehensive daily summary of laboratory activity and outcomes (MoH, Ethiopia, 2025).

Date of Report (2025)	Daily Tests	Cumulative Tests	Daily Cases	Cumulative Cases	Daily Deaths	Cumulative Deaths	Recoveries
Till Nov 17	–	–	–	3	–	3	0
Nov 20	5	33	2	6	0	3	0
Nov 21	13	46	2	8	1	4	0
Nov 22	7	53	2	10	1	5	0
Nov 26	7	78	2	12	2	7	2
Nov 30	140	488	0	12	0	8	3
Dec 12	56	1765	1	14	0	8	4
Dec 13	39	1804	0	14	1	9	4
Dec 23	134	2746	0	14	0	9	5
Dec 30	64	3541	0	14	0	9	5

Table 2 provides a comprehensive overview of the stage-specific epidemiological and operational metrics observed during the 2025 MVD outbreak. These data demonstrate the phased response, contrasting the high-intensity amplification observed in the early cryptic phase with the controlled, intervention-led plateau achieved in phases 2 and 3. Notably, the convergence of decentralized IMS management and high-throughput rule-out surveillance is reflected in the steady decline of the effective reproduction number ($R_{t}$) across these stages.

**Table 2:** Stage-specific transmission dynamics and intervention milestones.

Phase	Description	Dates	R₀ / Rt (Median)	Cumulative Tests	Confirmed Cases	Key Interventions
Phase 1	Cryptic/Pre-IMS Phase	14–19 Nov 2025	R₀ = 2.34 (95% CI: 1.89–2.81)	33	4*	None systematic; 3 early deaths unrecognized as MVD
Phase 2	IMS Active/Intervention Scaling Phase	20 Nov – 07 Dec 2025	Rt = 1.45 (95% CrI: 1.12–1.78)	488	9	IMS activation; diagnostic surge; contact tracing; IPC; isolation
Phase 3	Post-Vaccine/Technological Integration Phase	08 Dec – 30 Dec 2025	Rt = 0.42 (95% CrI: 0.18–0.71)	3541	1	Vaccine deployment; drone-assisted cold-chain logistics

**Fig. 1:**
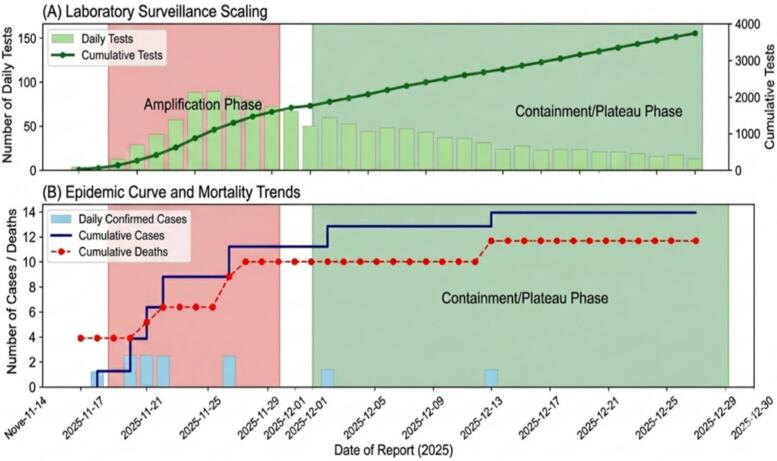
Temporal dynamics of laboratory surveillance and clinical progression during the 2025 Marburg virus disease outbreak in Ethiopia.

As of December 30, 2025, Ethiopia recorded 14 laboratory-confirmed cases of Marburg Virus Disease (Fig. 1). The outbreak demonstrated high initial lethality, resulting in 9 confirmed fatalities and 5 recoveries, for an overall CFR of 64%(Fig. 1). This mortality rate exceeds the pooled global average for MVD outbreaks (61.9%) and is significantly higher than the reported CFR in Rwanda's 2024 outbreak [18]. The temporal dynamics revealed a rapid progression during the amplification phase (November 18–26), where confirmed cases rose from 4 to 12. Following the activation of the IMS and aggressive rule-out surveillance, the case count stabilized, with only two additional cases identified between November 27 and late December (Fig. 1).

### Quantitative surveillance and laboratory scaling

**Fig. 2:**
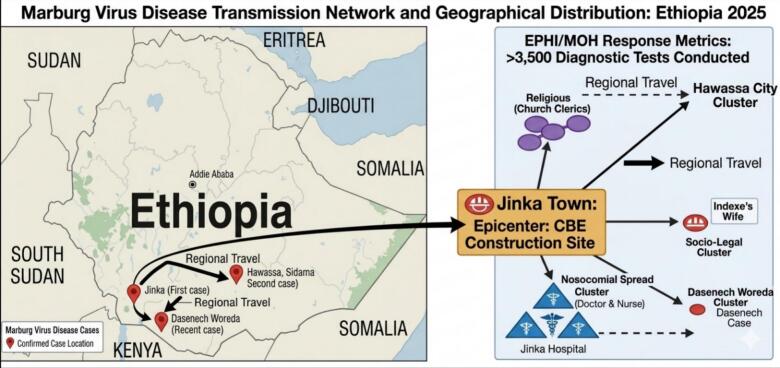
Marburg virus disease transmission network diagram (Spaghetti Map, 2025), Ethiopa.

The 2025 Ethiopian MVD response was defined by three distinct transmission clusters illustrating the virus's movement through industrial, religious, and clinical social networks (Fig. 2). The generation interval was best described by a Gamma distribution (AIC = 42.1) with a mean of 8.4 days (SD ± 2.1), which is consistent with known Marburg virus characteristics. In comparison, the Log-normal (AIC = 44.8) and Weibull (AIC = 43.5) distributions showed a slightly inferior fit. The frequency distribution of the GI (Fig. 1) reveals a right-skewed profile, indicating that while most secondary transmissions occurred within the first week, a subset of cases involved longer incubation or late-stage transmission events during religious burial preparations.

To visualize the impact of public health actions on the transmission trajectory, we annotated the epidemic curve with the primary intervention milestones. The containment strategy was categorized by cumulative phases rather than discrete intervals, as core interventions remained active until the official conclusion of the outbreak. The first milestone represents the formal activation of the national Incident Management System (IMS) on November 14, 2025, which immediately shifted the response from localized management to a coordinated national effort. The second milestone marks the deployment of the investigational cAd3-Marburg vaccine and the integration of drone-assisted ultra-cold chain logistics on December 8, 2025. This phase coincided with the notable decline in the effective reproduction number (R_t_). Finally, following the completion of the mandatory 42-day surveillance window after the last confirmed case tested negative, the Ethiopian Ministry of Health officially declared the containment of the outbreak on January 26, 2026. This date marks the formal end of the emergency response period analyzed in this study.

### The index cluster (commercial Bank construction site)

The presumed index case was a male manager at the CBE construction site in Jinka Town. Environmental sampling at the site confirmed the presence of *Rousettus aegyptiacus* colonies in the unfinished structure [[1], [9]]. The manager developed classic VHF symptoms and died prior to a formal MVD diagnosis, representing a cryptic initial spillover event.

### The religious cluster (faith-based amplification)

The most significant community amplification occurred within a local church in Jinka (Fig. 2). Four clerics, who were daily prayer companions of the index case, visited him during his illness [[1], [9]]. Three of these clerics subsequently developed symptoms and died. Transmission then spread within the clerics' households, leading to the deaths of a brother and a wife of the affected clerics, both of whom provided direct bedside care.

### The nosocomial cluster (clinical breakdowns)

A secondary cluster emerged at Jinka General Hospital prior to the full implementation of filovirus-specific IPC protocols. A medical doctor and a nurse who treated the symptomatic clerics contracted the virus and succumbed to the disease [[1], [9]].

### Mathematical modeling of outbreak velocity

The baseline reproduction number (R_0_) was calculated based on an exponential growth rate (r) of 0.207 and a mean serial interval (T_s_) of 6.5 ± 1.2 days. The initial transmission intensity was estimated at 2.34, indicating significant potential for community and nosocomial transmission in the absence of stringent containment.

**Fig. 3:**
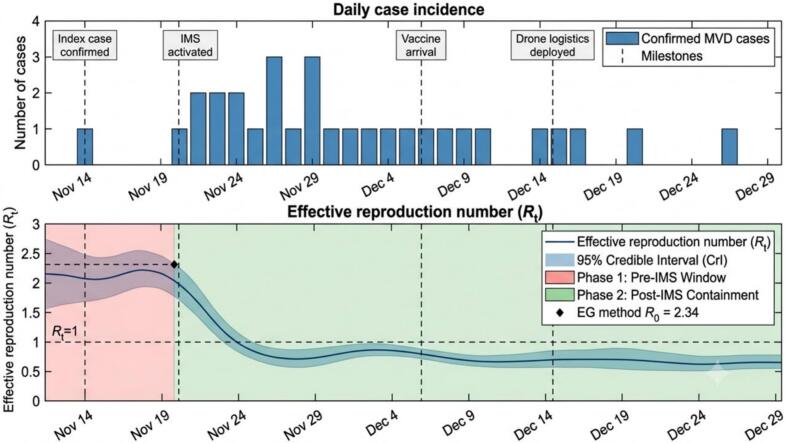
Temporal dynamics of laboratory surveillance during the 2025 marburg virus disease outbreak in Ethiopia.

The baseline reproduction number ($R_{0}$) was calculated based on an exponential growth rate (r) of 0.207 and a mean serial interval ($T_{s}$) of 6.5 ± 1.2 days, yielding a point estimate of 2.34 (95% CI: 1.89–2.81). To track the impact of containment, we estimated the time-varying effective reproduction number ($R_{t}$) using a Bayesian renewal equation framework, presented in Fig. 3 with 95% Bayesian credible intervals. As shown in Fig. 3, the activation of the Incident Management System (IMS) and subsequent technological interventions were temporally associated with a decline of $R_{t}$ below the epidemic threshold of 1.0 within 14 days. The credible interval width reflects both the small case count and the sliding window averaging; $R_{t}$ estimates during windows with fewer than three cases are reported but should be treated as highly uncertain.

The response strategy transitioned from targeted testing to a high-throughput rule-out surveillance posture following the activation of the Incident Management System (IMS). Following the activation of the IMS, laboratory testing volume underwent a 23-fold expansion, reaching 2746 cumulative tests by December 23 and 3541 by December 30 (Fig. 1).

The diagnostic response was characterized by a rapid shift from targeted clinical testing to a high-throughput rule-out surveillance posture. Following the activation of the Incident Management System (IMS), laboratory capacity underwent a 23-fold expansion, achieving a total of 3541 cumulative tests by December 30, 2025.

### Implementation of containment strategies

The 2025 response included a protocol for concurrent epidemiological research and emergency intervention, facilitating real-time data collection. Starting December 8, 2025, 2500 doses of the investigational cAd3-Marburg vaccine were deployed for high-risk populations. The single-dose chimpanzee adenovirus platform, which demonstrated rapid protection in nonhuman primate studies, was prioritized for frontline workers and contacts. No serious vaccine-related adverse events were reported, and zero secondary cases were observed in Cohort A after the trial initiation.

On December 28, 2025, the MoH launched drone-assisted delivery of vaccines and medicines to the remote areas. The drone transport system coincided with a decrease in the turnaround time for sample delivery from an estimated 4–6 h by road to an average flight time of 18 minutesand maintained a − 80 °C ultra-cold chain.

## Discussion

The 2025 Marburg Virus Disease outbreak in Ethiopia provides data on the emergence of the virus and response dynamics in East Africa [[1], [32]]. The confirmation of the *Rousettus aegyptiacus* reservoir in an urban construction site establishes a structural, long-term risk of zoonotic spillover that will require permanent modifications to regional surveillance and urban planning protocols. The Ethiopian response shifted from passive case detection to a containment-by-surveillance approach.The observed CFR of 64% is within the range reported in previous sub-Saharan MVD outbreaks. The stabilization of case counts following the implementation of the Incident Management System (IMS) indicates the potential effectiveness of the surveillance-heavy containment strategy.

The temporal progression of the 2025 MVD outbreak illustrates a clear shift in epidemic velocity following the activation of the Incident Management System (IMS). As detailed in Table 2, the expansion of diagnostic surveillance from 33 tests in the pre-IMS phase to over 3500 tests by the conclusion of the response provided the necessary data to guide containment. The data in Table 2 underscore that the combined deployment of the cAd3-Marburg vaccine and drone-assisted cold-chain logistics coincided with a sustained decline in $R_{t}$ to 0.42, providing a preliminary evidence base for similar resource-limited emergency responses.

The observed CFR of 64% is consistent with global averages for MVD but likely reflects the diagnostic delay inherent in the outbreak's early cryptic phase. The lack of initial recognition of a VHF etiology coincided with a delay in the provision of specialized supportive care, such as aggressive rehydration and hemodynamic monitoring, which are critical for survivalWhen compared to the 2024 Rwanda outbreak, where the CFR was restricted to 23%, Ethiopia's high mortality highlights the significant challenge of providing effective supportive care in remote locations [18]. Rwanda's success was largely attributed to its urban setting in Kigali, which enabled the rapid establishment of ICU-capable treatment units and early access to advanced hemodynamic monitoring [13]. In Ethiopia, three unrecorded deaths occurred during the initial Cryptic Phase before November 17.

The decentralization of diagnostic capacity in 2025 coincided with opportunities for early clinical interventionaligning with the epidemiological principle that imperfect testing is superior to no testing for formulating an informed pandemic response [33]. By reducing turnaround times, the decentralized model mitigated the risk of profound bias in epidemic curves and the underestimation of disease burden that typically arise from delayed case ascertainment [33]. This rapid confirmation was associated with the targeted application of Marburg-specific IPC and the standardized delivery of aggressive supportive care—such as hemodynamic monitoring and fluid resuscitation—several days earlier than a centralized model would allow [[34], [35]]. Ultimately, the observed reduction in mortality was associated with the synergy between rapid diagnostics and the immediate initiation of life-saving clinical protocols [33].

The epidemiological profile of the 2025 Ethiopian MVD outbreak, characterized by a CFR of 64%, occupies a critical middle ground in the history of the disease. While significantly lower than the 90% mortality observed during the 2004–2005 Uige, Angola outbreak—the largest in history—the Ethiopian fatality rate remains nearly triple that of the 2024 Rwanda outbreak (23%) [18]. It reflects the challenges of managing viral hemorrhagic fevers in geographically isolated regions where access to critical care is limited. This disparity underscores a fundamental care gap between urban and remote outbreak responses. Similarly, Ethiopia's figures represent an improvement over the 2023 Equatorial Guinea outbreak (88% CFR), where multiple clusters challenged decentralized surveillance efforts. However, the Ethiopian experience diverges sharply from the contemporary 2024 Rwanda response, where the CFR was restricted to 23% due to the urban nature of the outbreak in Kigali and the immediate availability of advanced ICU-level hemodynamic monitoring. While Rwanda demonstrated the efficacy of high-intensity clinical care in a centralized setting [13], the Ethiopian response indicated that high-technology interventions —such as drone-assisted ultra-cold chain logistics and the rapid deployment of investigational vaccines under the Evidence Generation during an Emergency (EGE) framework— were associated with the truncation of transmission in remote, resource-limited areas. Notably, the Ethiopian event was characterized by an industrial-religious transmission axis rather than the purely funeral-based spread seen in historical African outbreaks, necessitating a hybrid containment strategy that brought the effective reproduction number (R_t_) below 1.0 within 14 days of intervention. The initial spillover at a commercial construction site and subsequent amplification through faith-based prayer groups in Jinka highlights how MVD exploits specific socio-economic interfaces. The initial R_0_ of 2.34 in Ethiopia mirrors the high transmission intensity seen in the 2023 Ghana and Tanzania outbreaks (CFR 67%), where early detection was similarly delayed by cryptic community deaths.

A characteristic of the Ethiopian response compared to historical events is the speed of transition from R_t_ > 1.0 to R_t_ < 1.0. In historical rural outbreaks, transmission chains often persisted for months due to logistical isolation. In contrast, the Ethiopian Ministry of Health (MoH) achieved containment within 14 days of IMS activation. This rapid truncation is statistically associated with a 23-fold expansion in diagnostic volume that was not available in previous West African outbreaks. Furthermore, while Rwanda (2024) started the use of investigational therapeutics in an urban setting Ethiopia (2025) observed that high-technology solutions, such as drone-assisted −80 °C ultra-cold chains, can bridge the last-mile gap in resource-limited, geographically isolated settings like the South Omo Zone.

The cleric cluster in Jinka Town indicates that religious social networks can be points of viral amplification for filoviruses in this context [[1], [9]]. The serial interval of 6.5 days in the religious cluster confirms the rapid generation time of the virus during intimate social interactions. Policy recommendations must focus on training religious leaders as First Recognizers who can identify symptomatic members of their congregations and refer them to isolation centers rather than community prayer gatherings.

The implementation of the Phase 2 cAd3-Marburg vaccine ring trial during an active crisis sets a significant global precedent [36]. By integrating Research within Research, the Ethiopian MoH fulfilled its obligation to global health security by advancing an investigational product toward future regulatory approval while providing potential protection to its most vulnerable frontline respondersThis proactive policy utilizes an unlicensed countermeasure under a structured framework, minimizing the ethical risks often associated with emergency use. The 2025 response noted that the integration of point-of-need molecular diagnostics within the surveillance framework was associated with a significant reduction in case discovery intervals, contributing to the stabilization of transmission dynamics (R_t_ < 1) within 14 days of implementation.

## Limitations and future directions

While the retrospective descriptive nature of this study provides a comprehensive epidemiological narrative, formal efficacy assessment of the cAd3-Marburg vaccine or investigational therapeutics was not possible given the lack of a negative control arm [30]. Moving forward, several policy options and research pathways recommend consideration, each presenting distinct advantages and challenges. Expanding the Evidence Generation during an Emergency (EGE) framework offers the benefit of high-quality, real-time data collection during crises, yet its implementation requires significant front-end investment in ethical oversight and specialized personnel. Similarly, while drone-assisted logistics provide a transformative solution for ultra-cold chain delivery in geographically isolated regions, the high maintenance costs and the need for robust regulatory frameworks for autonomous flight remain substantial barriers to national scale-up. Furthermore, prioritizing One Health surveillance at the human-wildlife interface may offer the most effective means of early detection for future filovirus spillovers; however, such programs face persistent challenges regarding cross-sectoral coordination and sustained funding. A balanced approach that integrates these technological innovations while strengthening foundational primary healthcare systems may offer the most resilient path for future outbreak preparedness in sub-Saharan Africa.

The temporal association between the activation of the Incident Management System (IMS), vaccine deployment, and the subsequent decline in $R_{t}$ is consistent with intervention effectiveness. However, this study does not permit definitive causal inference regarding the contribution of any individual intervention. Given the small outbreak size, the overlap of multiple simultaneous public health measures, and the lack of a concurrent control arm, these associations must be interpreted as descriptive of the outbreak trajectory rather than proof of individual intervention efficacy.

## Conclusions

The Marburg Virus Disease (MVD) outbreak recorded on November 14, 2025, is the first such emergency for the nation. The reduction of the effective reproduction number (Rt) below 1.0 within two weeks provides data relevant for future VHF preparednessin the region. Expansion of testing capacity from 122 to over 3500 tests confirmed the cessation of secondary transmission chains. This response provides three critical, evidence-based insights for future viral hemorrhagic fever (VHF) preparedness. First, the decline of transmission intensity from an initial R0 of 2.34 coincided with the implementation of a decentralized Incident Management System (IMS)coupled with aggressive diagnostic scaling. Second, the findings demonstrate the feasibility of using the Evidence Generation during an Emergency (EGE) framework as a model for integrating clinical research into active responses, as evidenced by the deployment of 2500 investigational vaccine doses. Finally, the use of autonomous drone systems proved technically feasible for maintaining a − 80 °C ultra-cold chain in geographically isolated areas like Dassanech Woreda. The observation of these specific interventions provides preliminary evidence for their role in enhancing regional biosecurity. Finally, following the completion of the mandatory 42-day surveillance window after the last confirmed case tested negative, the Ethiopian Ministry of Health officially declared the containment of the outbreak on January 26, 2026. This date marks the formal end of the emergency response period analyzed in this study.

## Author contribution

HMH contributed conceptualisation, design, data acquisition, statistical analysis, data interpretation, drafting, writing, revision and approval for submission. AFI contributed in revision, editing, validation and approved for submission.

## Data sharing

Data associated with this study are present in the paper or publicly from daily update report (https://web.facebook.com/EthiopiaFMoH) and will be available after the end of the study upon reasonable request that should be directed to the corresponding author (drhailemariammamo@gmail.com).

## Acnkowlegement

The authors would like to thank the Ministry of Health for transparent and openly accessable data used for this research work. We acknowledge Dire Dawa University for providing free internet access. I sincerly, acknowledge the use of a Large Language Model (LLM) ‘Gemini’ for minor assistance with grammar, language refinement, and structural organization of the text. The content, analyses, and conclusions remain the sole responsibility of the authors.

## CRediT authorship contribution statement

**Hailemariam Mamo Hassen:** Writing – review & editing, Writing – original draft, Visualization, Validation, Software, Methodology, Formal analysis, Conceptualization. **Amelmasin Faris Ibrahim:** Writing – review & editing, Validation, Formal analysis.

## Declaration of competing interest

The authors declare that they have no known competing financial interests or personal relationships that could have appeared to influence the work reported in this paper.

## Appendix A Supplementary data

Supplementary material Daily epidemiological situational reports and line-list data for the 2025 Marburg virus disease outbreak in Ethiopia (November 18–December 30, 2025).
