# Supplementary material for: Epidemiology and containment of the first Marburg virus disease outbreak in Ethiopia in 2025: A retrospective descriptive study
Source: Glob Epidemiol. 2026 Jul 1;12:100276. doi: 10.1016/j.gloepi.2026.100276 (PMC13352164; doi:10.1016/j.gloepi.2026.100276)

*Marburg virus diseases daily report Ministry of Health Ethiopia from 18 November 2025 to 14 December 2025*


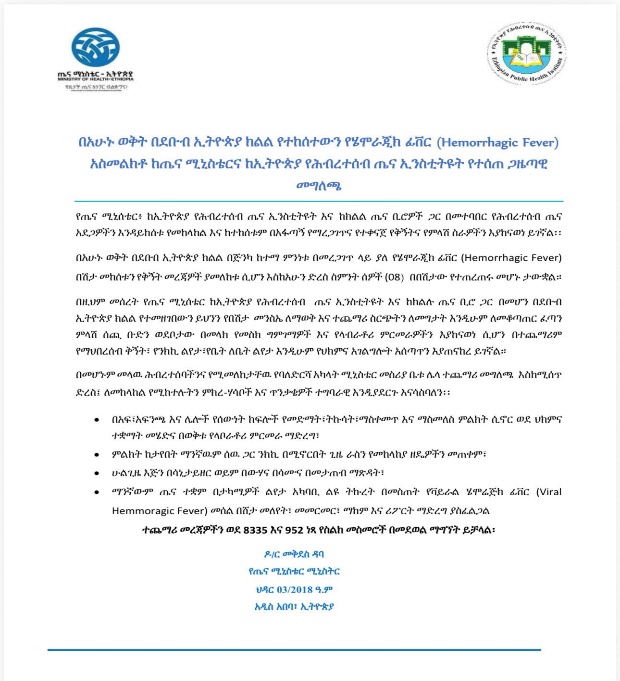

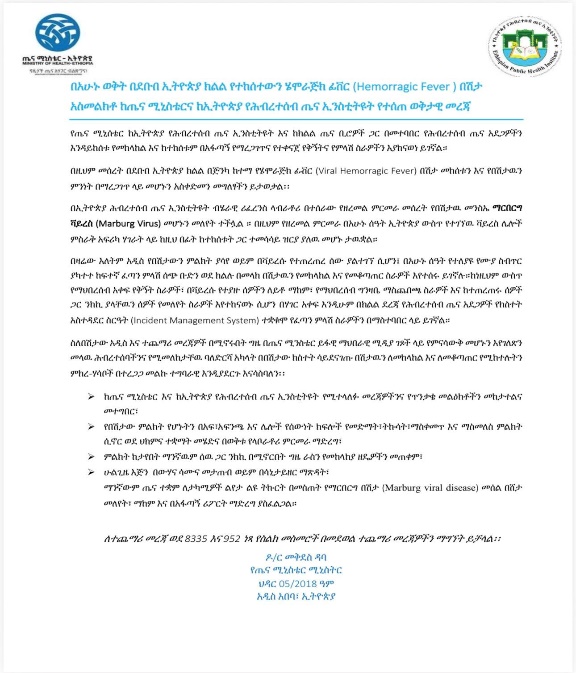


**Report on MOH Press Release for Research Article**

Initial announcement and confirmation of Marburg Virus Disease (MVD). Status update outlining the response activation (Incident Management System) and continuing public health measures

The Ministry of Health (MOH) of Ethiopia, in collaboration with the Ethiopian Public Health Institute (EPHI), issued two crucial press releases (dated 03/2018 E.C. and 05/2018 E.C.) regarding a confirmed outbreak of **Marburg Virus Disease (MVD)**, initially identified as a suspected Viral Hemorrhagic Fever, in the South Ethiopia Regional State.

The first release confirmed the presence of the Marburg virus following laboratory testing by EPHI, establishing the public health threat. In response to the threat, release the MOH immediately activated a comprehensive **Incident Management System (IMS)** to coordinate the national response. Key intervention strategies included enhanced disease surveillance, rigorous contact tracing, strict infection prevention and control (IPC) measures in health facilities, and the immediate isolation and treatment of confirmed cases. Furthermore, the MOH engaged in extensive risk communication, mobilizing communities and urging the public to report suspected cases immediately via dedicated toll-free numbers (8335 or 952) and advising strict preventative measures, such as avoiding contact with bats, wild animals (e.g., monkeys), and the bodily fluids of sick or deceased individuals. The ongoing response effort is being conducted in collaboration with international partners, including the World Health Organization (WHO) and Africa CDC.


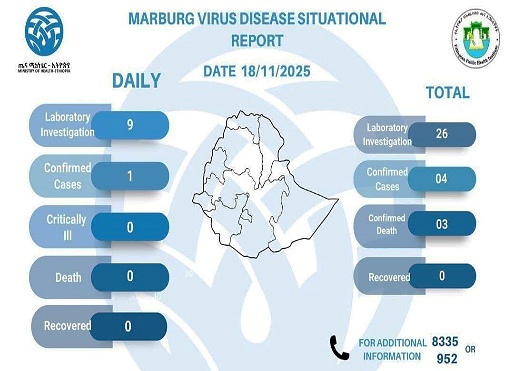


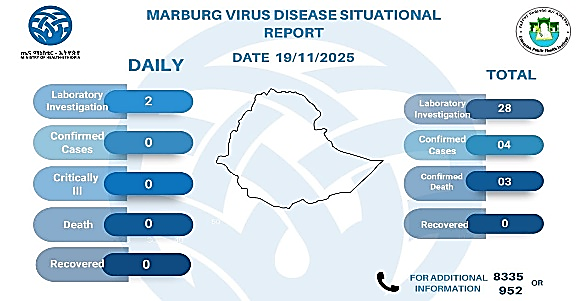

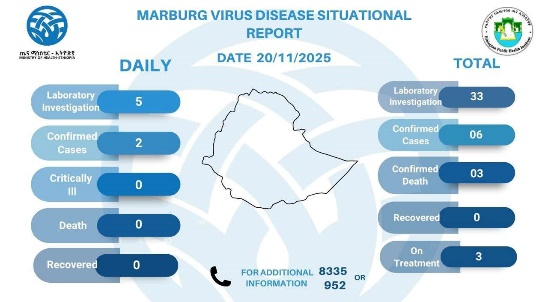


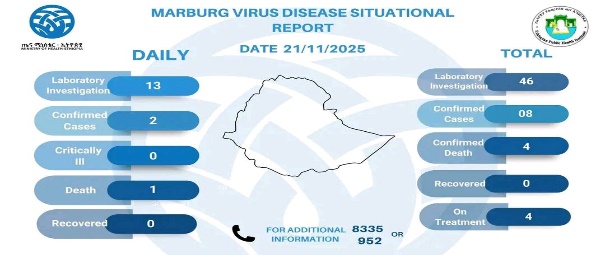

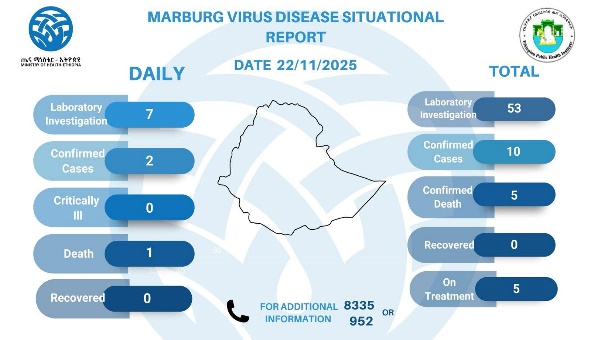

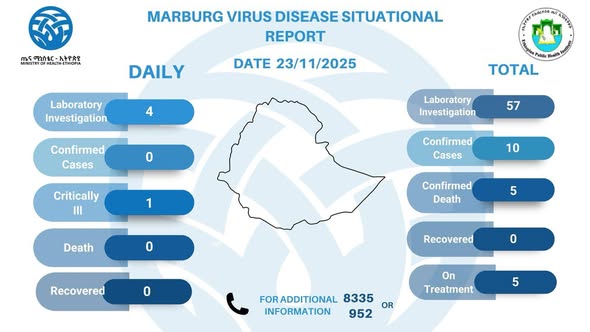


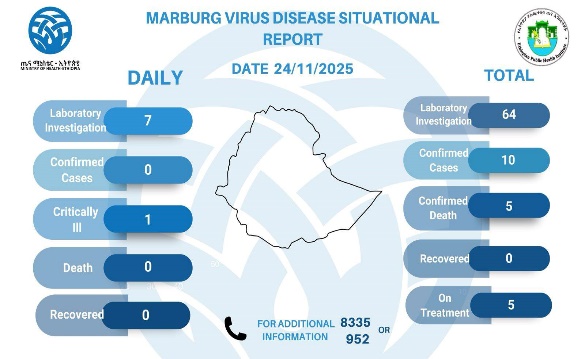

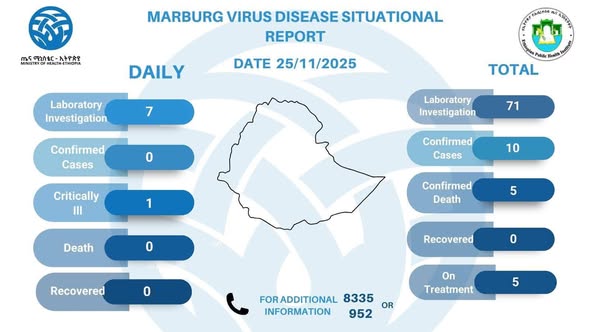

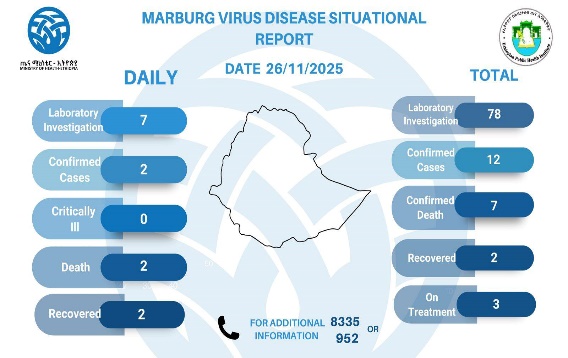


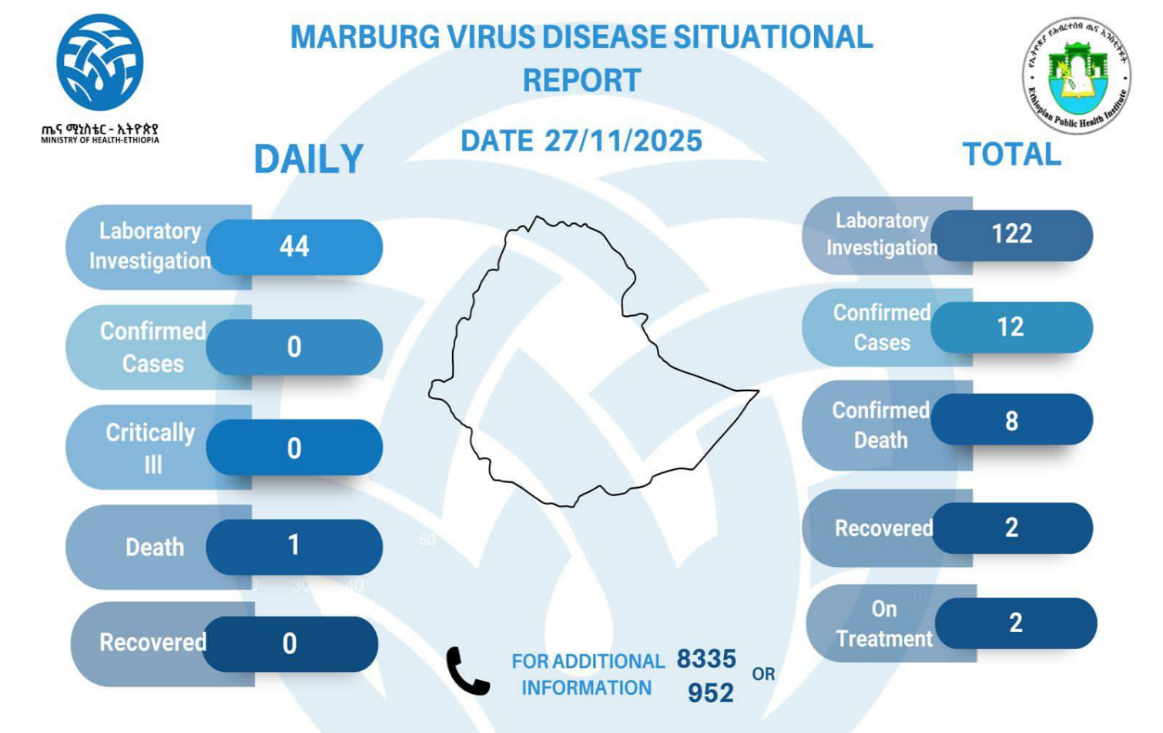


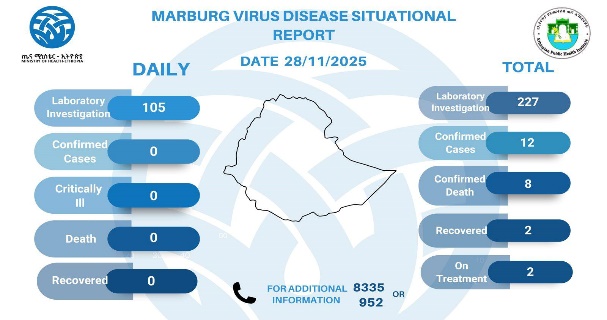

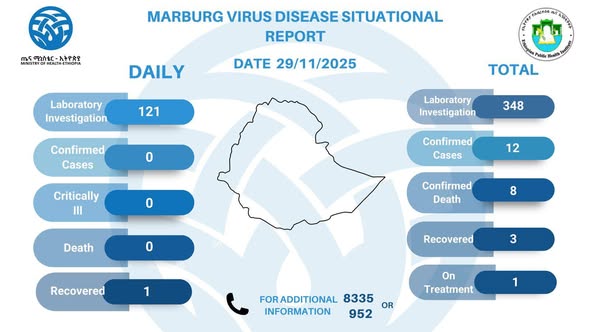

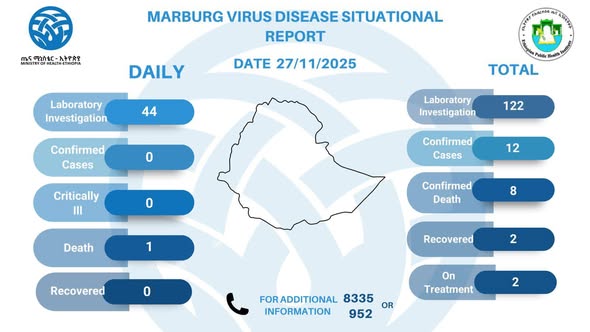


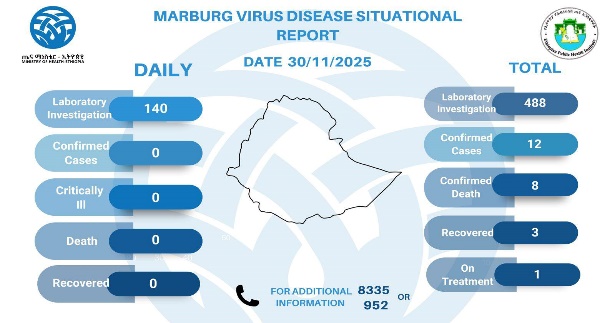

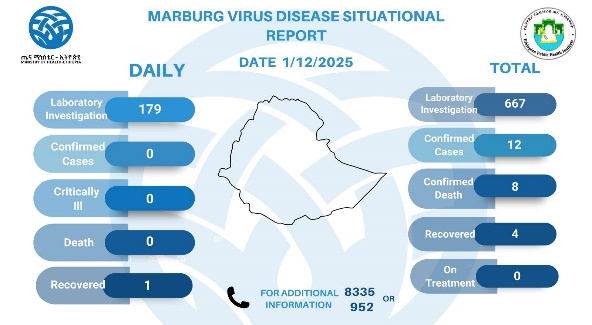

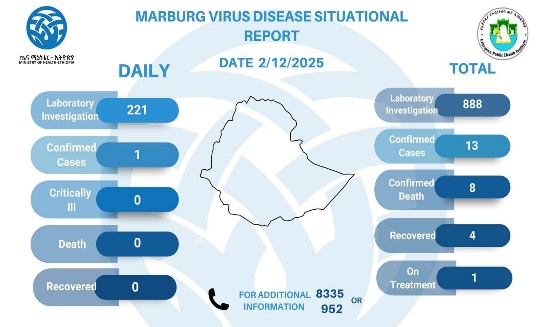


**14**


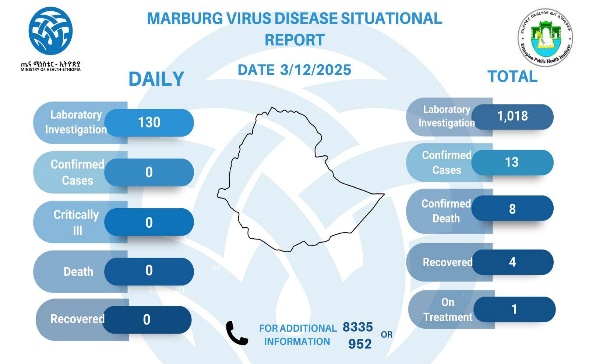

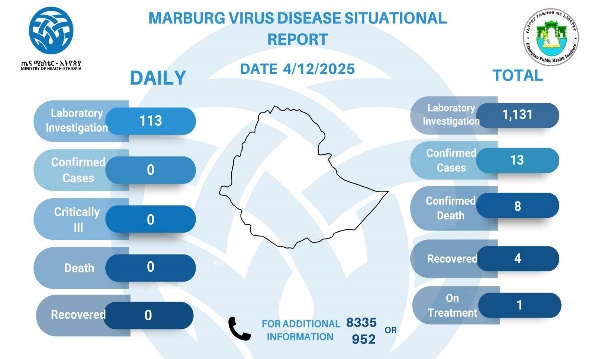

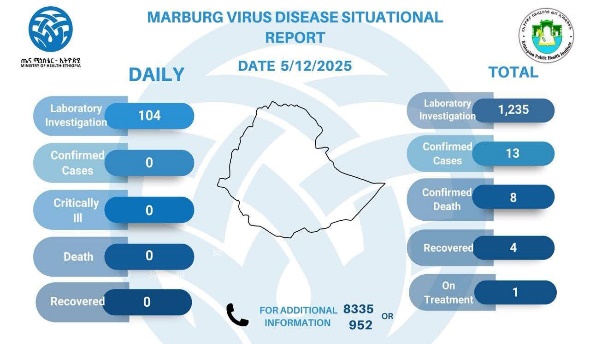


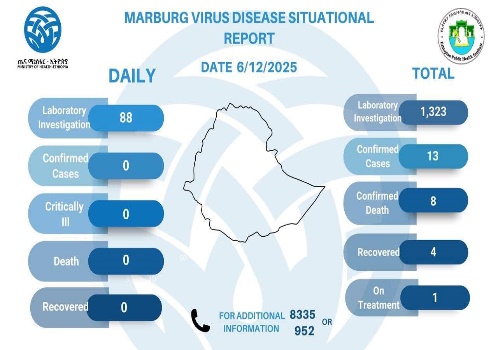

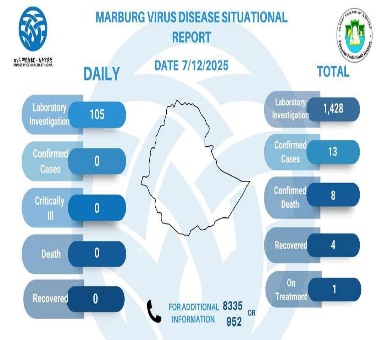

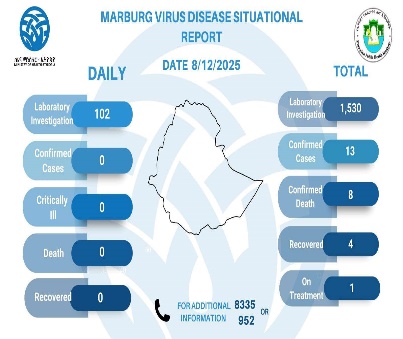

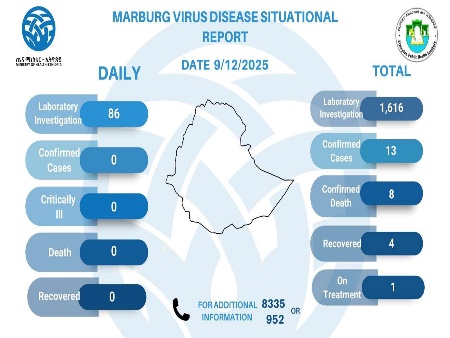


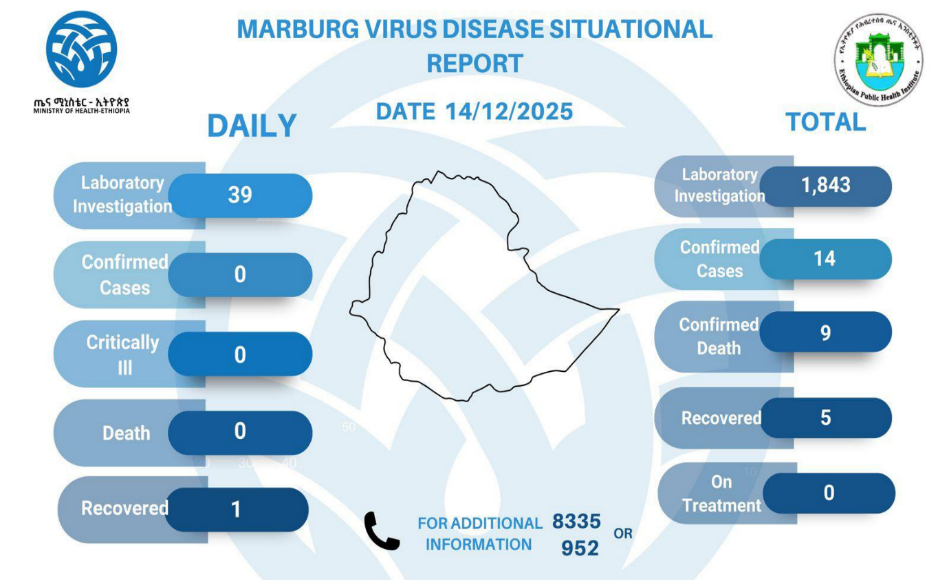


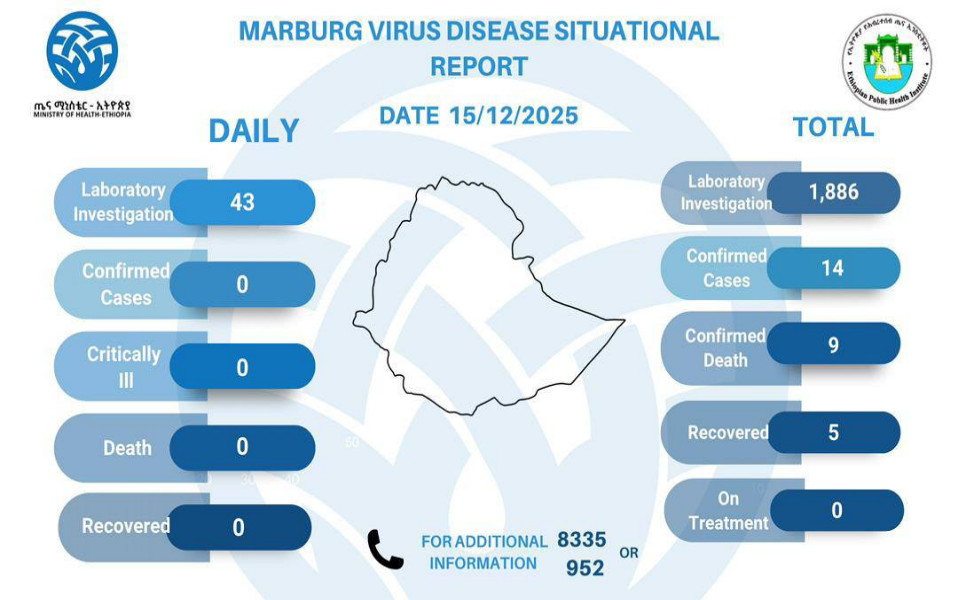


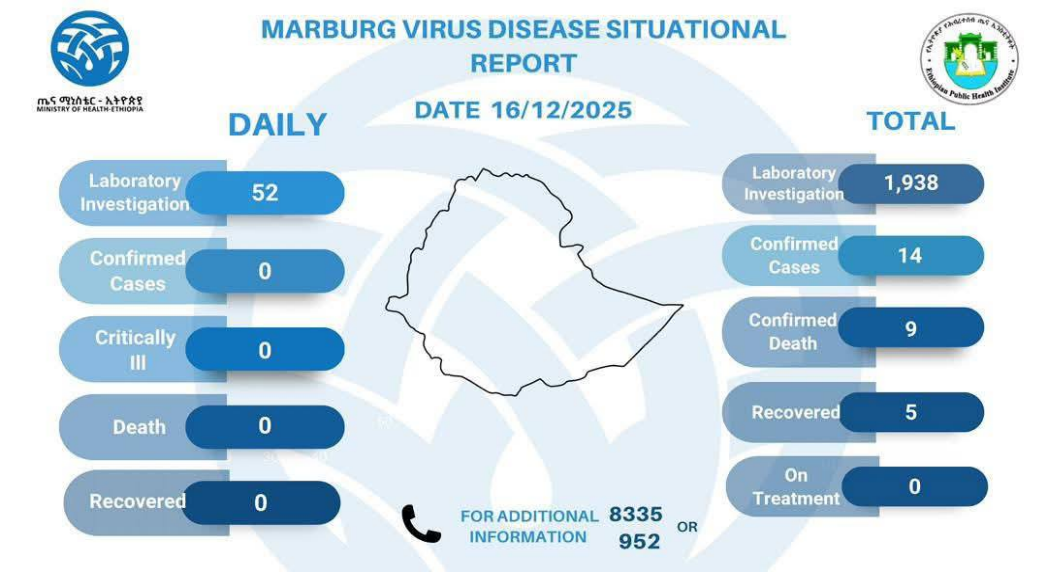


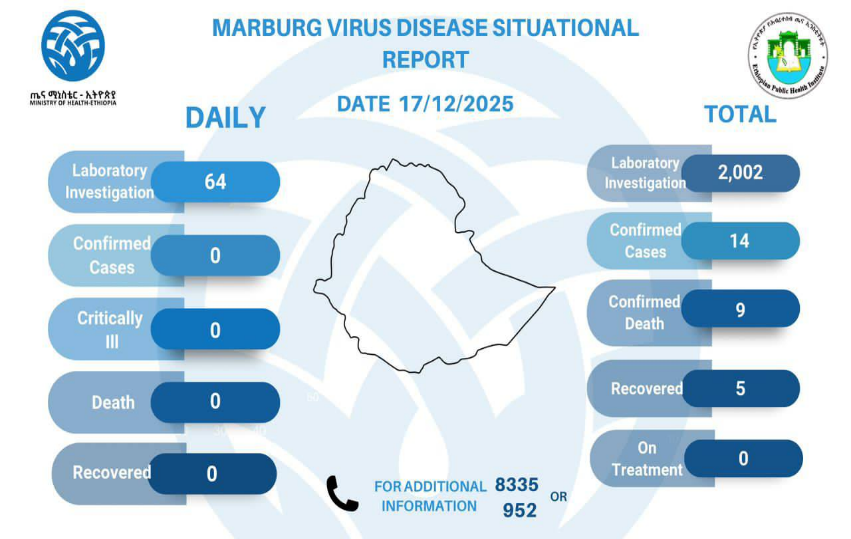


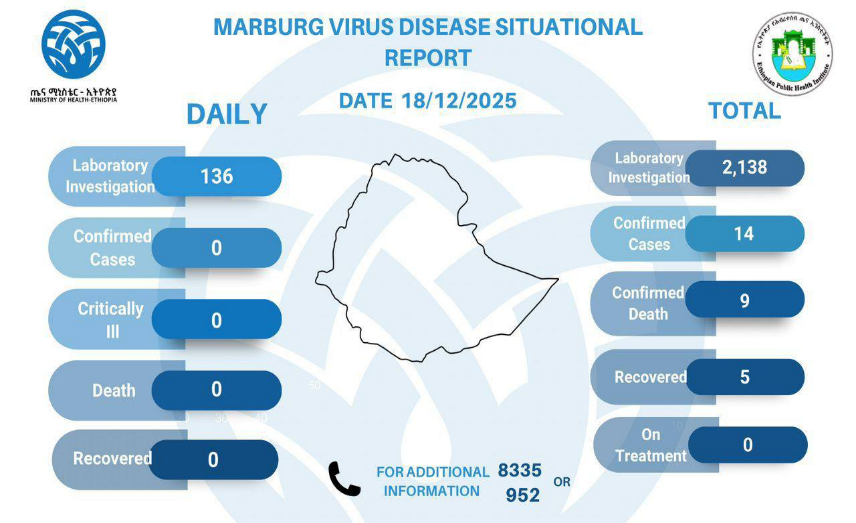


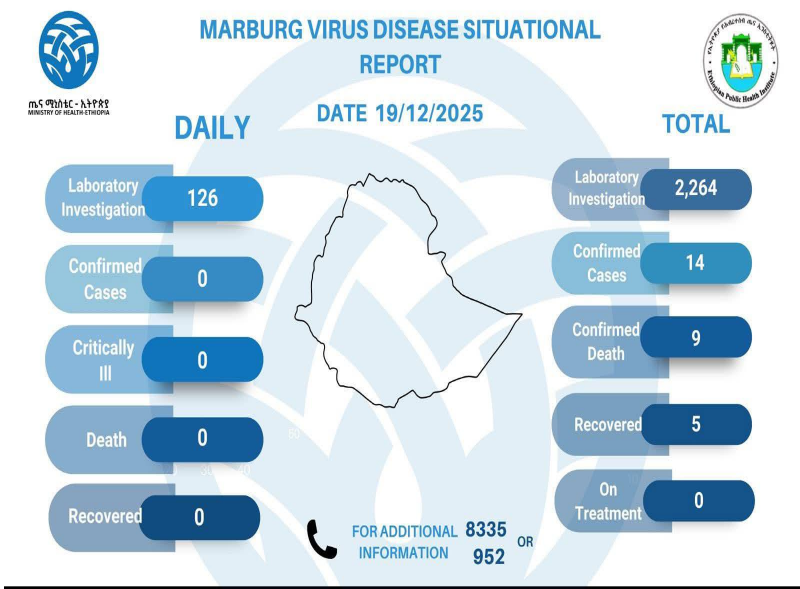


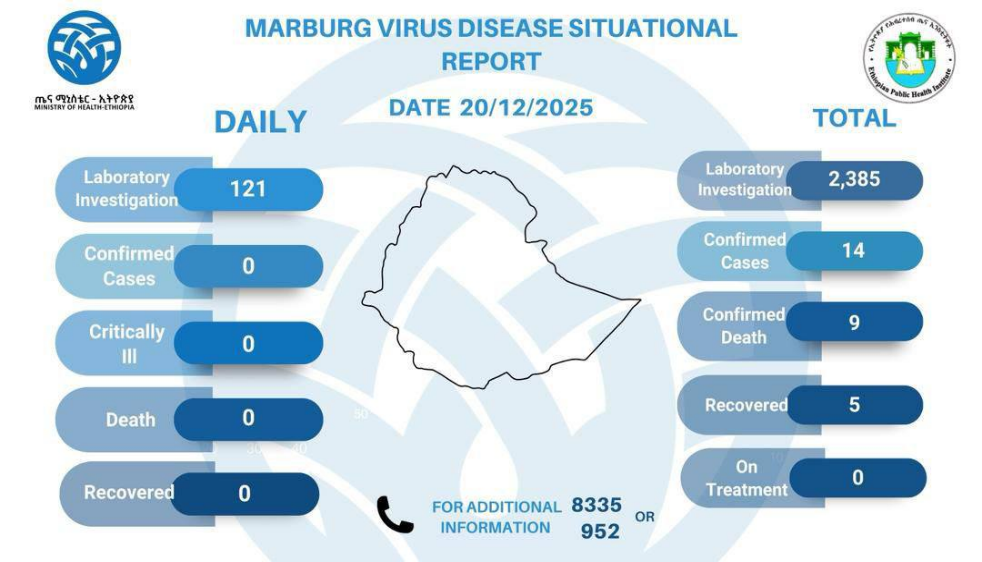


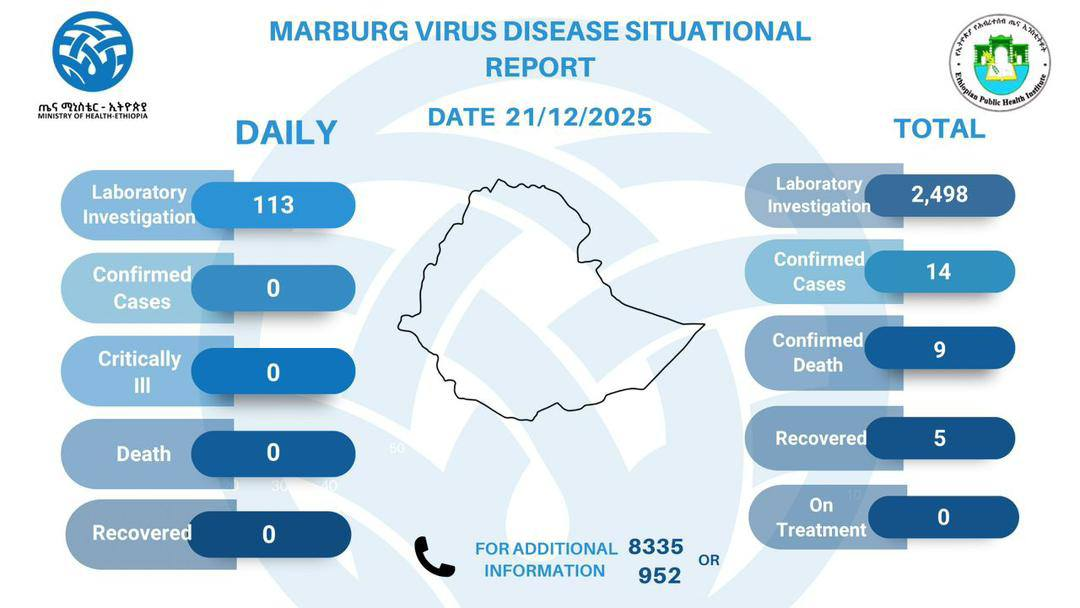


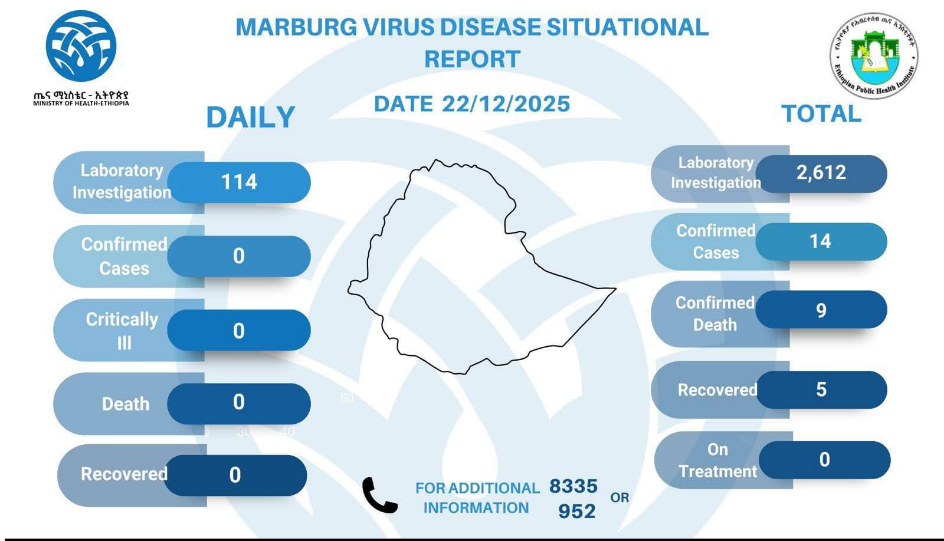


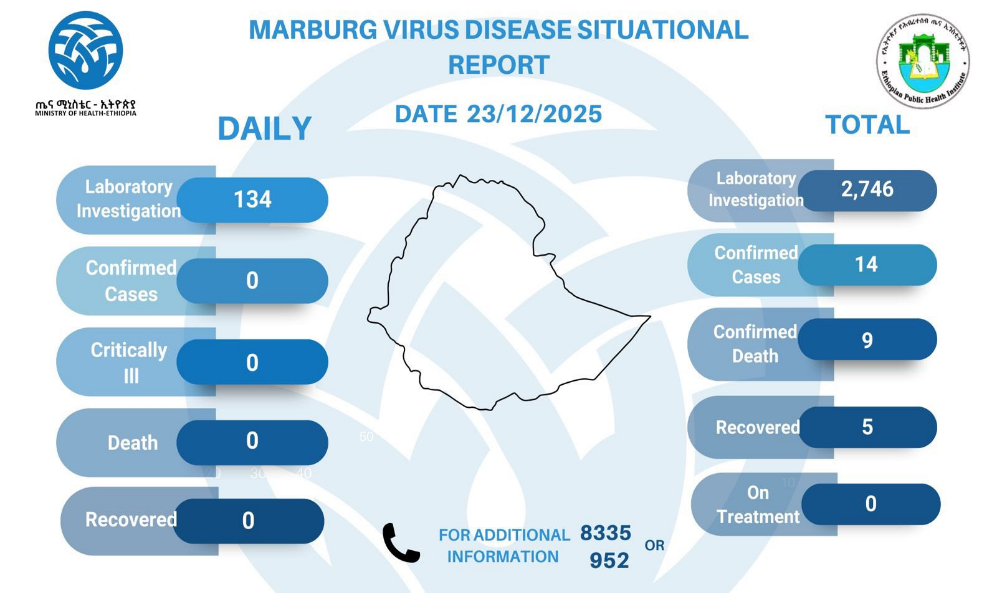


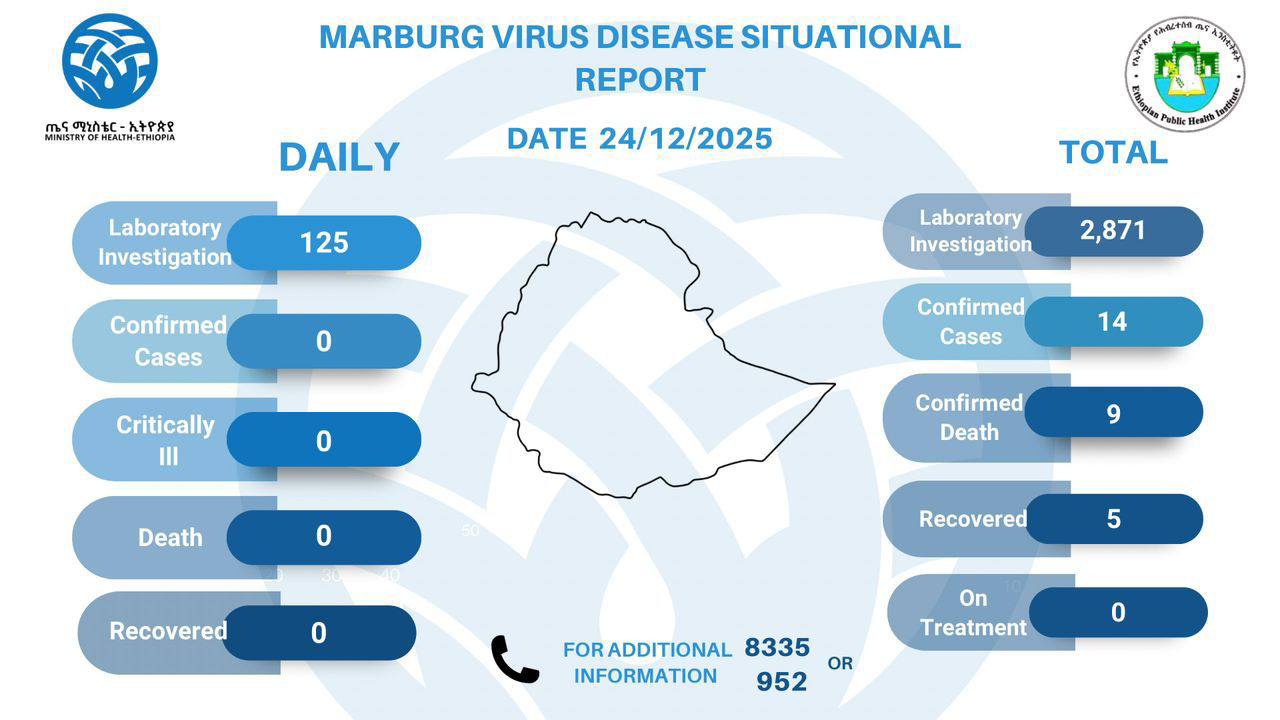

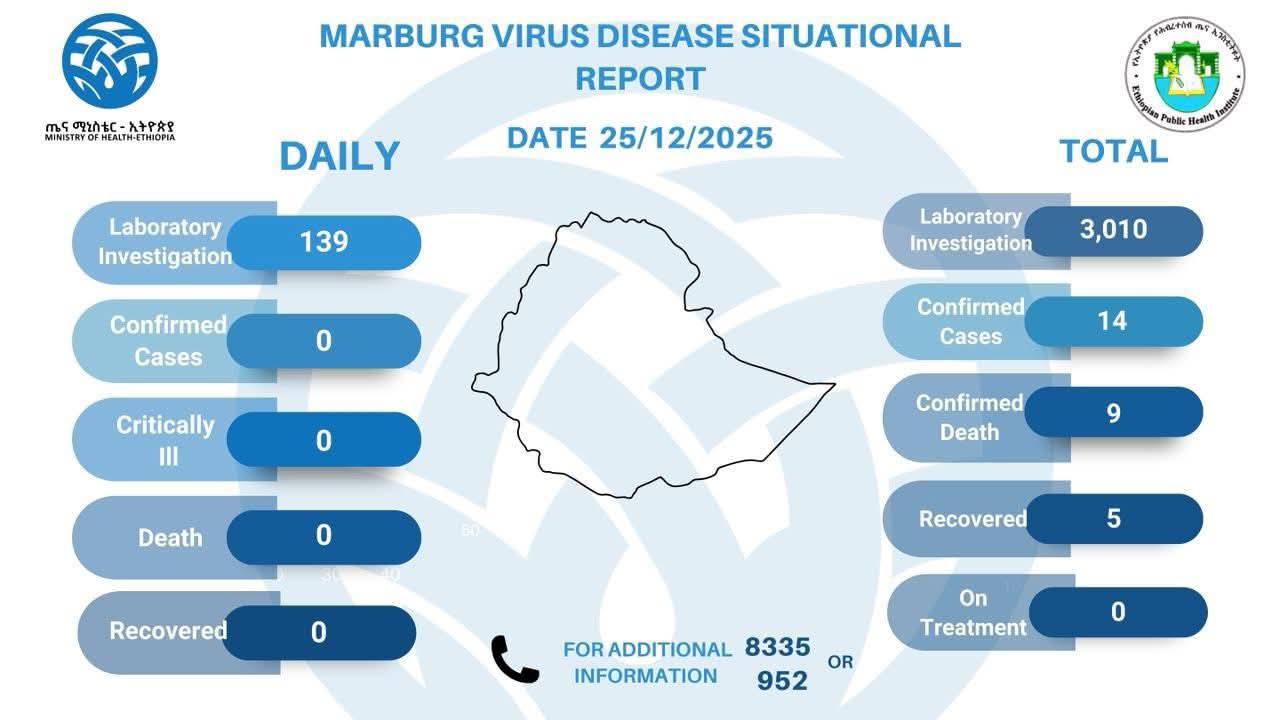

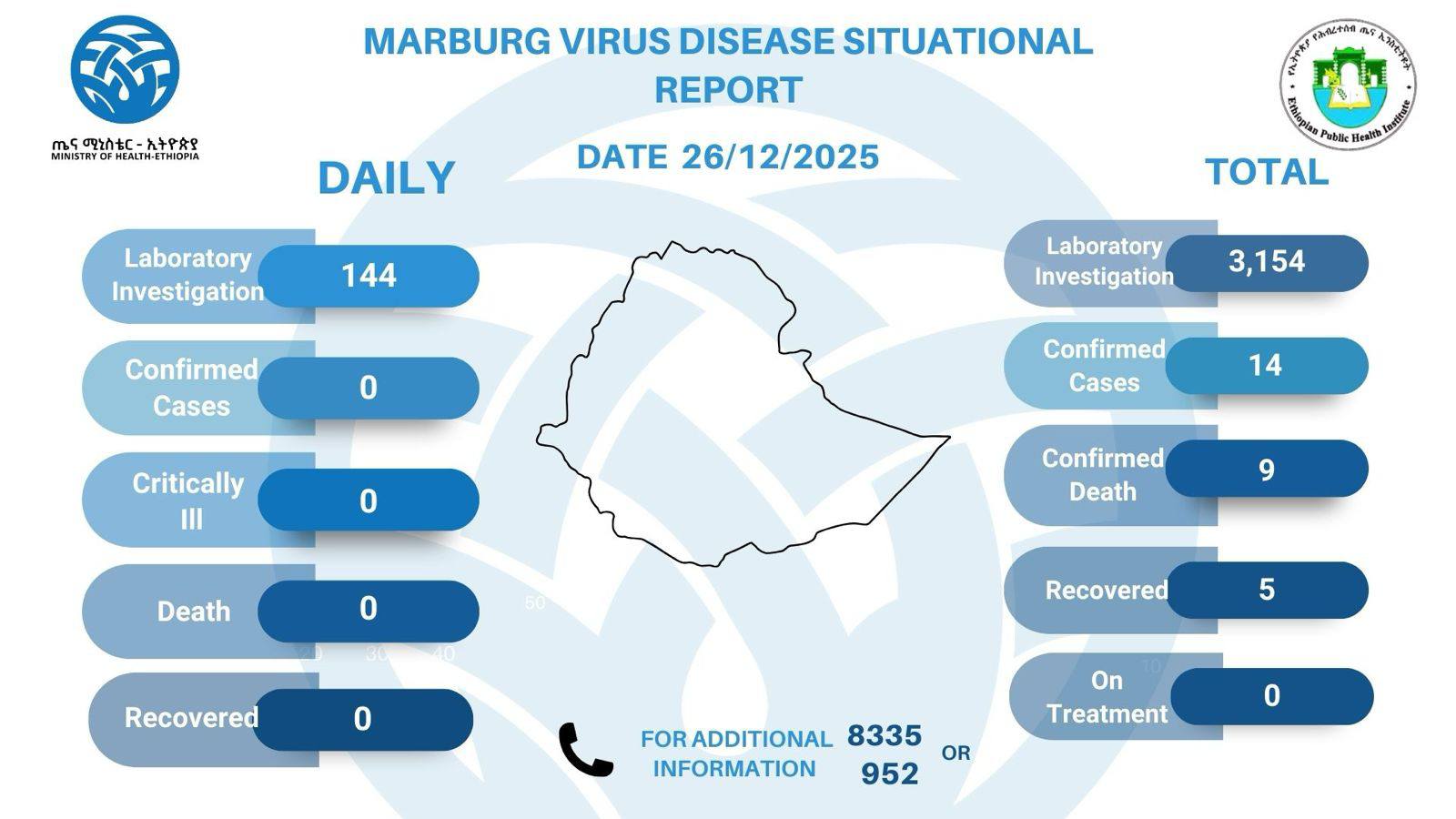

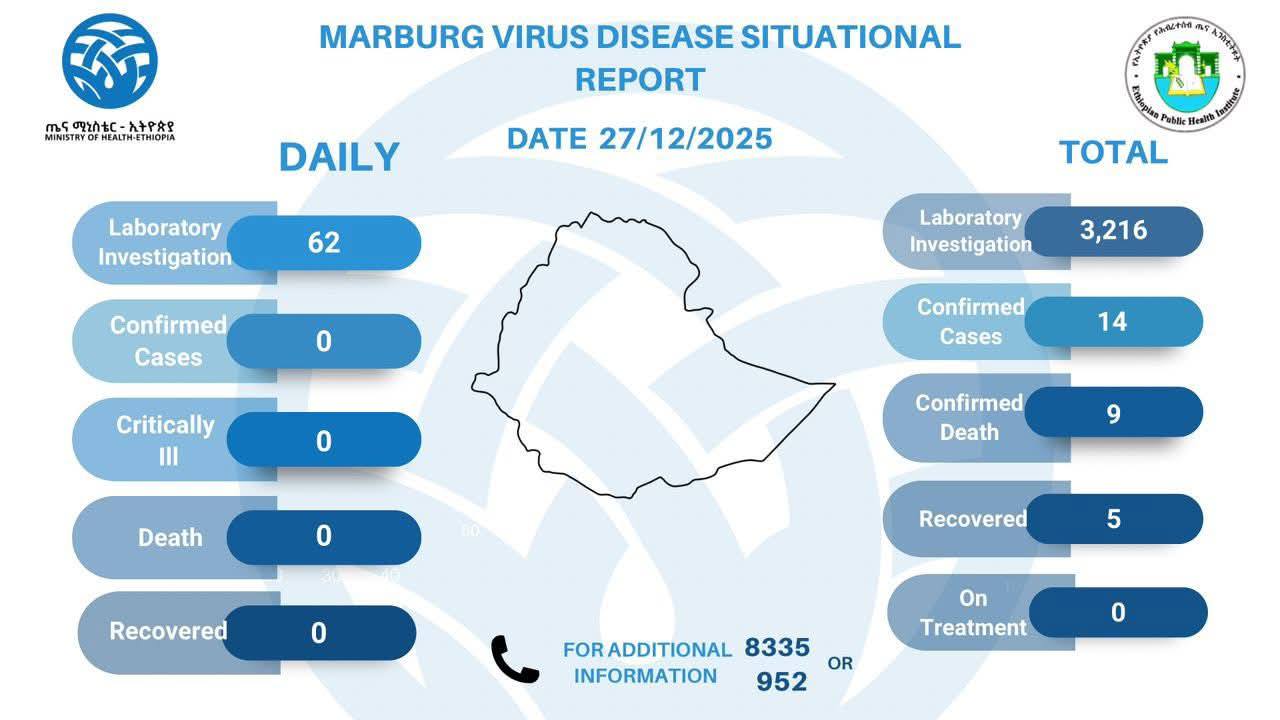


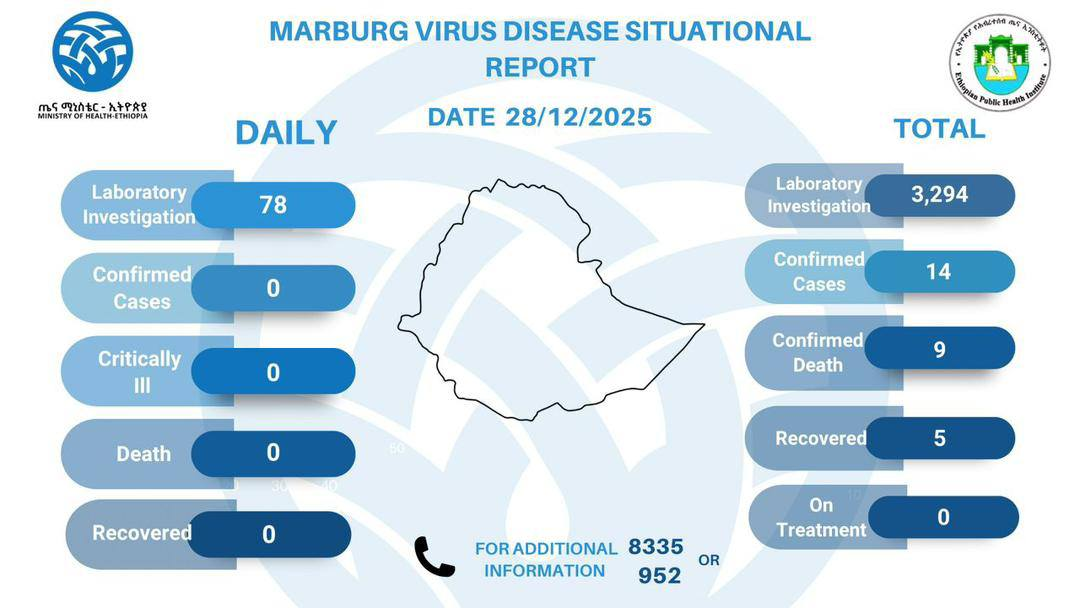


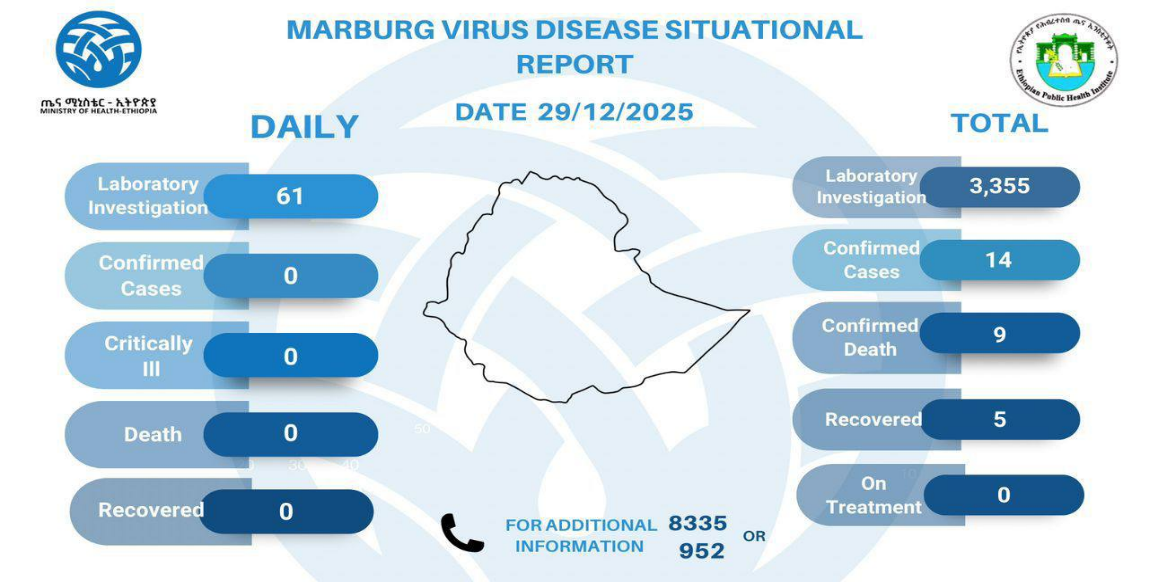


| **Date of report by MoH** | **Daily suspected Laboratory tests** | **Cumulative Daily suspected Laboratory tests** | **Daily Confirmed Cases** | **Daily Confirmed Deaths** | **Cumulative Confirmed Cases** | **Cumulative Confirmed Deaths** | **Cumulative Recovered** | **Cumulative On Treatment** |
| --- | --- | --- | --- | --- | --- | --- | --- | --- |
| Till 17/11/2025 |  |  |  |  | 3 | 3 | 0 |  |
| 18/11/2025 |  | 26 | 1 | 0 | 4 | 3 | 0 |  |
| 19/11/2025 | 2.00 | 28 | 0 | 0 | 4 | 3 | 0 |  |
| 20/11/2025 | 5.00 | 33 | 2 | 0 | 6 | 3 | 0 | 3 |
| 21/11/2025 | 13.00 | 46 | 2 | 1 | 8 | 4 | 0 | 4 |
| 22/11/2025 | 7.00 | 53 | 2 | 1 | 10 | 5 | 0 | 5 |
| 23/11/2025 | 4.00 | 57 | 0 | 0 | 10 | 5 | 0 | 5 |
| 24/11/2025 | 7.00 | 64 | 0 | 0 | 10 | 5 | 0 | 5 |
| 25/11/2025 | 7.00 | 71 | 0 | 0 | 10 | 5 | 0 | 5 |
| 26/11/2025 | 7.00 | 78 | 2 | 2 | 12 | 7 | 2 | 3 |
| 27/11/2025 | 44.00 | 122 | 0 | 1 | 12 | 8 | 2 | 2 |
| 28/11/2025 | 105.00 | 227 | 0 | 0 | 12 | 8 | 2 | 2 |
| 29/11/2025 | 121.00 | 348 | 0 | 0 | 12 | 8 | 3 | 1 |
| 30/11/2025 | 140.00 | 488 | 0 | 0 | 12 | 8 | 3 | 1 |
| 01/12/2025 | 179.00 | 667 | 0 | 0 | 12 | 8 | 4 | 0 |
| 02/12/2025 | 221.00 | 888 | 1 | 0 | 13 | 8 | 4 | 1 |
| 03/12/2025 | 130.00 | 1018 | 0 | 0 | 13 | 8 | 4 | 1 |
| 04/12/2025 | 113.00 | 1131 | 0 | 0 | 13 | 8 | 4 | 1 |
| 05/12/2025 | 104.00 | 1235 | 0 | 0 | 13 | 8 | 4 | 1 |
| 06/12/2025 | 88.00 | 1323 | 0 | 0 | 13 | 8 | 4 | 1 |
| 07/12/2025 | 105.00 | 1428 | 0 | 0 | 13 | 8 | 4 | 1 |
| 08/12/2025 | 102.00 | 1530 | 0 | 0 | 13 | 8 | 4 | 1 |
| 09/12/2025 | 86.00 | 1616 | 0 | 0 | 13 | 8 | 4 | 1 |
| 10/12/2025 | 60.00 | 1676 | 0 | 0 | 13 | 8 | 4 | 1 |
| 11/12/2025 | 33.00 | 1709 | 0 | 0 | 13 | 8 | 4 | 1 |
| 12/12/2025 | 56.00 | 1765 | 1 | 0 | 14 | 9 | 4 | 2 |
| 13/12/2025 | 39.00 | 1804 | 0 | 1 | 14 | 9 | 4 | 1 |
| 14/12/2025 | 39.00 | 1843 | 0 | 0 | 14 | 9 | 5 | 0 |
| 15/12/2025 | 43.00 | 1886 | 0 | 0 | 14 | 9 | 5 | 0 |
| 16/12/2025 | 52.00 | 1938 | 0 | 0 | 14 | 9 | 5 | 0 |
| 17/12/2025 | 64.00 | 2002 | 0 | 0 | 14 | 9 | 5 | 0 |
| 18/12/2025 | 136.00 | 2138 | 0 | 0 | 14 | 9 | 5 | 0 |
| 19/12/2025 | 126.00 | 2264.00 | 0 | 0 | 14 | 9 | 5 | 0 |
| 20/12/2025 | 121.00 | 2385.00 | 0 | 0 | 14 | 9 | 5 | 0 |
| 21/12/2025 | 113.00 | 2498.00 | 0 | 0 | 14 | 9 | 5 | 0 |
| 22/12/2025 | 114.00 | 2812.00 | 0 | 0 | 14 | 9 | 5 | 0 |
| 23/12/2025 | 134.00 | 2746.00 | 0 | 0 | 14 | 9 | 5 | 0 |
| 24/12/2025 | 125.00 | 2871.00 | 0 | 0 | 14 | 9 | 5 | 0 |
| 25/12/2025 | 139.00 | 3019.00 | 0 | 0 | 14 | 9 | 5 | 0 |
| 26/12/2025 | 144.00 | 3158.00 | 0 | 0 | 14 | 9 | 5 | 0 |
| 27/12/2025 | 62.00 | 3210.00 | 0 | 0 | 14 | 9 | 5 | 0 |
| 28/12/2025 | 78.00 | 3360.00 | 0 | 0 | 14 | 9 | 5 | 0 |
| 29/12/2025 | 61.00 | 3477.00 | 0 | 0 | 14 | 9 | 5 | 0 |
| 30/12/2025 | 64.00 | 3541.00 | 0 | 0 | 14 | 9 | 5 | 0 |

**The Ministry of Health launched drone-assisted vaccine distribution ()**

**The Ministry of Health, in collaboration with the Ethiopian Information Network Security Administration, officially launched drone-assisted vaccine distribution in Dasenech Wereda.**

The Ministry of Health, in collaboration with the Information Network Security Administration (INSA), officially launched a drone-based delivery service today to transport life-saving medicines and vaccines to areas at risk of the Marburg virus.

As part of the Ministry's rapid response measures to curb the spread of the outbreak and save the lives of citizens in remote areas, a drone-assisted vaccine distribution was successfully carried out today in Dasenech Wereda, South Ethiopia Region.

Given the remote geography of Dasenech Wereda, a trip that would have taken several hours by vehicle was completed in just a few minutes using drones. The vaccines were delivered while maintaining a cold chain of –80°C, and administration to the local community has commenced.

This initiative practically demonstrates that the Ministry of Health and the Ethiopian Pharmaceutical Supply Service (EPSS) have elevated their capacity for early pandemic prevention and control by utilizing technological advancements.

Furthermore, it serves as a significant indicator that Ethiopia has begun utilizing its internal capacity and technology to combat outbreaks. This drone-based medical delivery service will continue to be strengthened in coordination with relevant stakeholders.

be strengthened in coordination with relevant stakeholders.


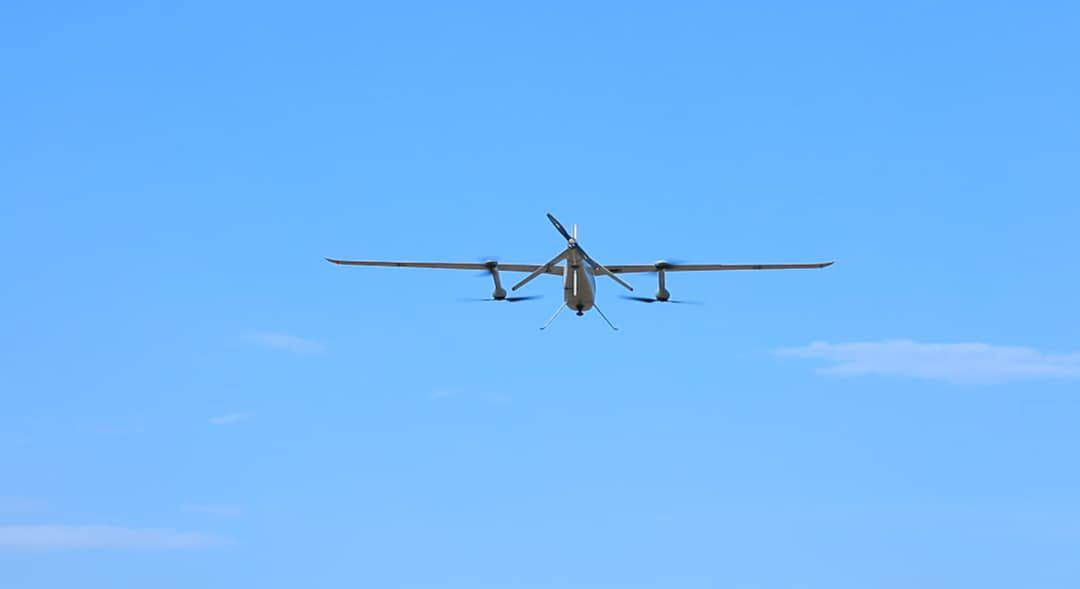

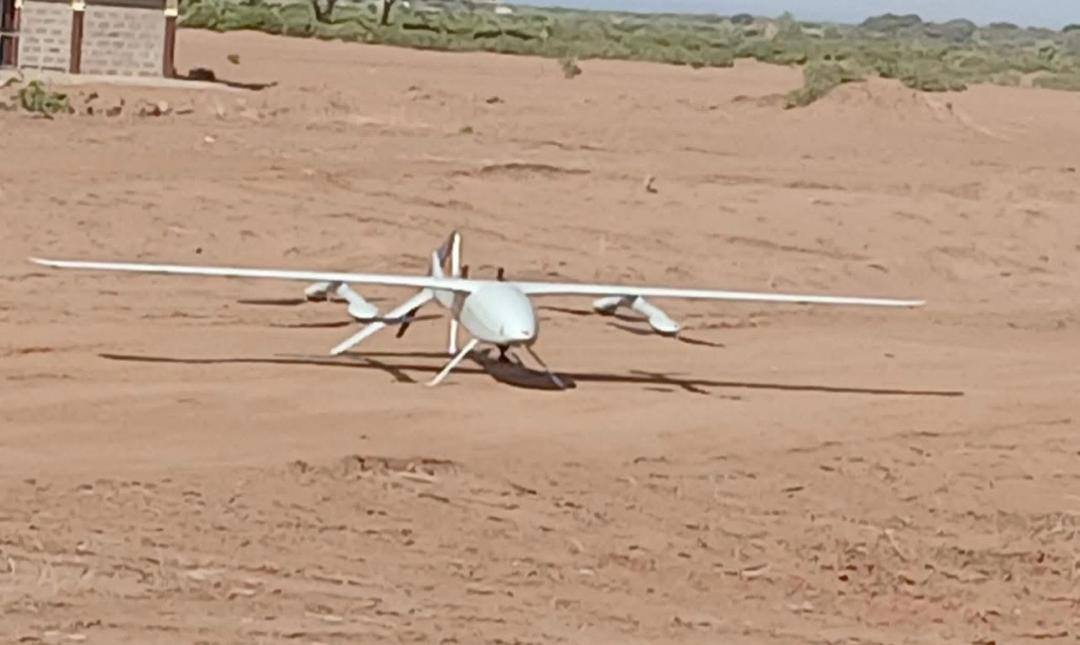


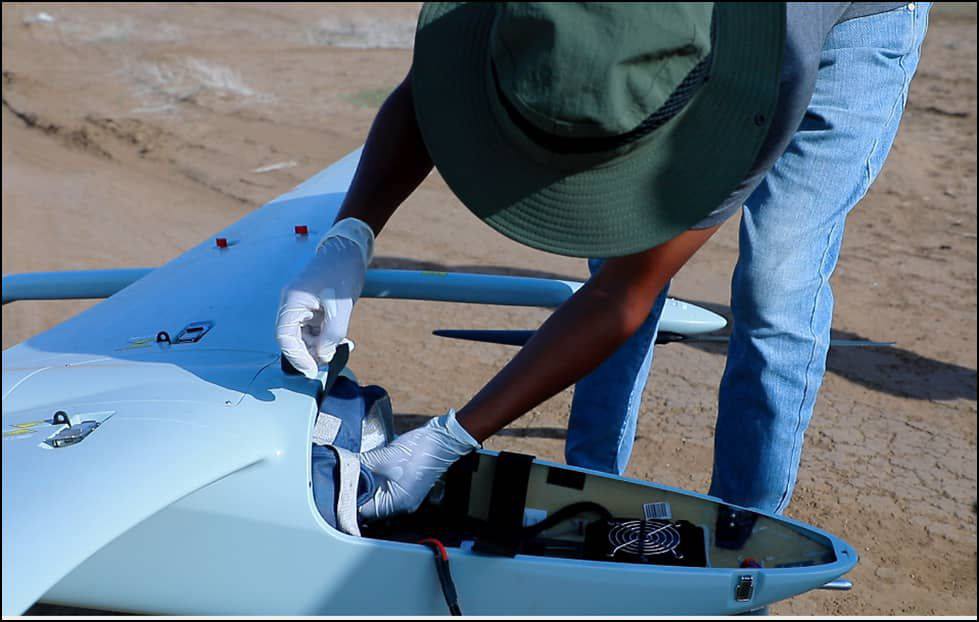


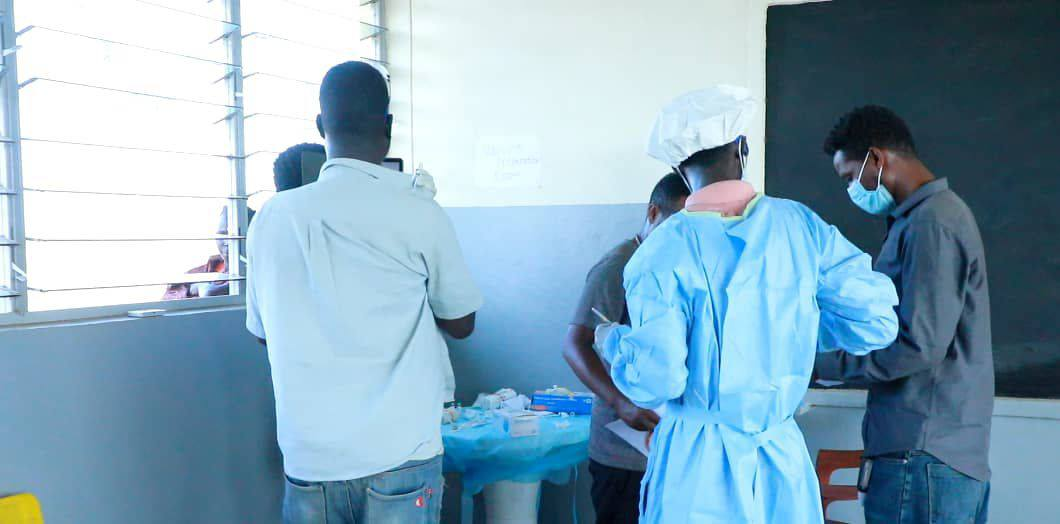

Supplement: Supplementary file 1 — Supplementary material Daily epidemiological situational reports and line-list data for the 2025 Marburg virus disease outbreak in Ethiopia (November 18–December 30, 2025). [file mmc1.docx]
